# Perspectives for Photocatalytic Decomposition of Environmental Pollutants on Photoactive Particles of Soil Minerals

**DOI:** 10.3390/ma17163975

**Published:** 2024-08-09

**Authors:** Agnieszka Sosnowska, Kinga I. Hęclik, Joanna B. Kisała, Monika Celuch, Dariusz Pogocki

**Affiliations:** 1 Department of Landscape Architecture, Institute of Environmental Engineering, Warsaw University of Life Sciences—SGGW, Nowoursynowska 166, 02-787 Warsaw, Poland; agnieszka_sosnowska@sggw.edu.pl; 2 Institute of Biology, College of Natural Sciences, University of Rzeszow, Rejtana 16C, 35-959 Rzeszow, Poland; kinga.i.heclik@wp.pl (K.I.H.); jkisala@ur.edu.pl (J.B.K.); 3 Łukasiewicz Research Network—Warsaw Institute of Technology, Duchnicka 3, 01-796 Warsaw, Poland; monika.celuch@wit.lukasiewicz.gov.pl; 4 Institute of Nuclear Chemistry and Technology, Dorodna 16, 03-195 Warsaw, Poland

**Keywords:** photocatalysis, semiconductors, sunlight, soil minerals, micro-pollutants, pathogens, environmental decontamination

## Abstract

The literature shows that both in laboratory and in industrial conditions, the photocatalytic oxidation method copes quite well with degradation of most environmental toxins and pathogenic microorganisms. However, the effective utilization of photocatalytic processes for environmental decontamination and disinfection requires significant technological advancement in both the area of semiconductor material synthesis and its application. Here, we focused on the presence and “photocatalytic capability” of photocatalysts among soil minerals and their potential contributions to the environmental decontamination in vitro and in vivo. Reactions caused by sunlight on the soil surface are involved in its normal redox activity, taking part also in the soil decontamination. However, their importance for decontamination in vivo cannot be overstated, due to the diversity of soils on the Earth, which is caused by the environmental conditions, such as climate, parent material, relief, vegetation, etc. The sunlight-induced reactions are just a part of complicated soil chemistry processes dependent on a plethora of environmental determinates. The multiplicity of affecting factors, which we tried to sketch from the perspective of chemists and environmental scientists, makes us rather skeptical about the effectiveness of the photocatalytic decontamination in vivo. On the other hand, there is a huge potential of the soils as the alternative and probably cheaper source of useful photocatalytic materials of unique properties. In our opinion, establishing collaboration between experts from different disciplines is the most crucial opportunity, as well as a challenge, for the advancement of photocatalysis.

## 1. Introduction: The Basics of Photocatalysis and Strategies to Enhance Its Effectiveness

The subject of relationships between pollution and infectious disease has become a hot topic among scientists even prior to the recent COVID-19 pandemic years [1]. The pandemic itself has sparked a surge in interest in implementing measures to eliminate toxins, viruses and other pathogens from the environment. Since the advent of the photocatalysis, the method has been proposed for “self-cleaning” solutions, maintenance of clean surfaces, and depolluting applications allowing the removal of inorganic and organic pollutants present in heavily polluted environments. The decomposition and destruction of pollutants are caused by processes involving highly reactive oxidative species (ROS) generated on the surface of semiconductors when exposed to light [2,3,4,5,6,7,8,9].

For the record, the semiconductors are characterized by a filled valence band (VB) and an empty conduction band (CB) [10,11]. Thus, upon light illumination by photons of energy higher than the band gap (*E*_BG_) [12], electrons are excited and promoted into CB ($e_{\mathrm{CB}}^{-}$), leaving holes ($h_{\mathrm{VB}}^{+}$) in VB. The photo-generated $e_{\mathrm{CB}}^{-}$-$h_{\mathrm{VB}}^{+}$ pairs will then migrate to the surface of photocatalyst, where, in contact with the aqueous environment and oxygen, they produce ROS [3,13]. (A less topic-oriented reader can easily find details and in-depth knowledge on semiconductors and photocatalysis in many basic books and articles, of which we will mention only a few [3,4,8,10,14,15,16,17,18,19,20,21,22,23,24,25,26,27,28,29,30,31]. The basic issues related to environmental pollution and related chemistry problems can be found in many textbooks and monographs [32,33,34,35]).

It is necessary to highlight here that four essential elements for photocatalytic oxidation should be present: (i) a photocatalyst, stable under given pH and temperature conditions; (ii) sunlight; (iii) atmospheric oxygen; and (iv) humidity. The sufficiently small *E*_BG_ of the photocatalyst allows the absorption of natural sunlight, whereas atmospheric oxygen and humidity allow the formation and transport of ROS out of the photocatalyst surface.

The most common photocatalysts are represented by metal oxides or sulphides such as TiO_2_, ZnO, ZnS, Fe_2_O_3_, and SnO_2_ or less frequently used but intensively tested Fe_3_O_4_, MoS_2_, and CdS [25,36,37,38,39,40,41,42]. The photocatalysts are natural or synthesized in laboratory conditions on a technical scale [43,44,45].

A plethora of experimental and theoretical works conducted over the last twenty years or so demonstrate the effort put into improving the efficiency of photocatalysts. The effort to obtain desired changes in the characteristics of photocatalysis usually goes in two directions: (i) utilization of a broader light absorption spectrum for enhanced quantum yield of ROS and (ii) an increase in the reducing properties of $e_{\mathrm{CB}}^{-}$, with the goal of being able to reduce water to molecular hydrogen.

It is worth emphasizing that the manipulation of the dimensions and shape of a semiconductor particle allows change in the photocatalysts’ characteristics [10,46,47,48,49]. Another popular and quite effective way to “tune” *E*_BG_, the Fermi level (i.e., the reduction potentials of $e_{\mathrm{CB}}^{-}$ and $h_{\mathrm{VB}}^{+}$), the molar absorption coefficient (ε), and other characteristics of the material is by the engineering that introduces dopants and other defects (physical, including porosity, twin boundaries, and cracks, and chemical, including vacancies and interstitials) into the structure of the semiconductor [50,51,52,53,54,55,56,57,58,59]. Thus, under certain conditions, even high dielectric constant oxides like ZrO_2_ can be utilized to build binary oxide photocatalysts [60,61,62,63,64].

Due to the material engineering such as the incorporation of various “impurity” elements and crystal lattice defects, microstrains, and dislocations, with size and shape control, the materials could possess “the visible-light-driven photocatalytic activity” (VLD) [53,65,66,67,68,69]. (However, extended defects can provoke the recombination of $e_{\mathrm{CB}}^{-}$-$h_{\mathrm{VB}}^{+}$ pairs, which causes reduced yield of ROS generation [22,52]).

The particles of naturally occurring minerals have unique chemical compositions that contain a main component and many other trace amounts of elements. They are formed through complex biogeochemical processes that are hardly understood and, despite many attempts, are very difficult to imitate in the laboratory conditions [70,71,72,73,74,75].

This directs interest to natural photoactive minerals, with their possible sources related to shallow deposits in the Earth’s crust and in the soil. Our focus was on the minerals found in the outer layers of the Earth’s crust and their potential contributions to the environmental decontamination in vitro and in vivo. We tried to explore these issues from the perspectives of chemists and environmental scientists, especially because we have not found attempts in the literature to describe the soil as a medium in which photocatalytic decontamination may occur.

## 2. Photoactalytic Activity of Earth’s Lithosphere Minerals: Natural Semiconductors Present in the Crust and Their Capabilities to Initiate Redox Reactions

Semiconducting minerals are ubiquitous on Earth, most of which are common mineral phases located near the Earth’s surface: oxides (e.g., *rutile*, *limonite*, *hematite*, and *goethite*) and sulphides (e.g., *sphalerite*, *greenockite*, and *pyrite*) [76]. Xu and Schoonen have reviewed about fifty kinds of semiconducting metal oxides and sulphide minerals, as shown in Table 1 [26].

The data gathered above (Table 1) can be discussed in a very simplified and condensed way: Minerals of *E*_BG_ ≤ ca. 3.3 eV (380 nm) possess VLD. The oxide minerals are strong photo-oxidation catalysts in aqueous solutions but are limited in their reducing power. The majority of metal oxide semiconductors have valence band edges (*E*_VB_) in the range 1 to 3 V above the reduction potential of $H_{2}O$ (relative to the electrochemical SHE scale [77,78]), and energies for conduction band edges (*E*_CB_) are close to or less negative than the reduction potential of $H_{2}O$. More specifically, the electron generated in CB can reduce the substance if *E*_CB_ is more negative than the reduction potential of the substance (reactant) (*E*r) (i.e., *E*_CB_ < *E*r). Similarly, the $h_{\mathrm{VB}}^{+}$ generated in the valence band can oxidize a substance if its reduction potential is lower than the *E*_VB_ of the semiconductor (i.e., *E*_VB_ > *E*_r_). One should note that none of the minerals (which are presented in Table 1), upon the light exposure, can promote electrons to CB, generating $e_{\mathrm{CB}}^{-}$, which is able to reduce H_2_O and become the hydrated electron ($e_{\mathrm{aq}}^{-}$) of reduction potential $E_{\mathrm{aq}/e_{\mathrm{aq}}^{•-}}^{⊖}$ equal to −2.87 V [81]. Such electrons cannot escape into and migrate throughout the solution; thus, its reactions are limited to the immediate vicinity of the surface (see discussion in [82]). It should be noted that if *E*_CB_ < 0 V, $e_{\mathrm{CB}}^{-}$ can reduce the H_3_O^+^ cation to molecular hydrogen (H_2_) since the reduction potential of SHE ($E_{H^{+}{+/H}_{2}}^{⊖}$) is by definition equal to 0 V [77,78]. On the other hand, in the majority of minerals in Table 1, the light-induced promotion of electrons to CB creates $h_{\mathrm{VB}}^{+}$ with *E*_VB_ higher than EOH/OH−•⊖ = 1.9 V [81], allowing oxidation of an ^-^OH anion to an ^•^OH radical. As one can see, for a much smaller number of minerals, $h_{\mathrm{VB}}^{+}$ has a reduction potential higher than EOH,H+•/H2O⊖ = 2.73 V [81], allowing oxidation of H_2_O in a neutral or acidic solution. Please note that non-transition metal sulphides generally have both *E*_CB_ and *E*_VB_ of higher energies than metal oxides; therefore, $h_{\mathrm{VB}}^{+}$ here are less oxidizing, but $e_{\mathrm{CB}}^{-}$ are rarely reducing, while most transition metal sulphides are characterized by small *E*_BG_ (<1 eV, ca. 1340 nm) with both the oxidizing power $h_{\mathrm{VB}}^{+}$ and the reducing power of $e_{\mathrm{CB}}^{-}$ lower than those of non-transition metal sulphides.

Additionally, one has to bear in mind that both *E*_CB_ and *E*_VB_ are pH-dependent since the ion balance on the mineral’s surface is affected by the pH. Thus, the oxidizing power of $h_{\mathrm{VB}}^{+}$ and the reducing power of $e_{\mathrm{CB}}^{-}$ will also depend on the pH. For semiconducting metal oxides, the *E*_CB_ and *E*_VB_ vary with pH, following the Nernstian relation (1) [26,83]

(1) $$E_{\mathrm{CB}(\mathrm{orVB})}=E_{\mathrm{CB}(\mathrm{orVB})}^{⊖}+2.303\frac{RT}{F\times ({\mathrm{pH}}_{\mathrm{zpc}}-\mathrm{pH})},$$

where $E_{\mathrm{CB}(\mathrm{orVB})}^{⊖}$ is the potential at the pH of the zero point of charge (pH_zpc_), and the net adsorbed charge within the Helmholtz double layer [80] is zero. Thus, the pH has to be taken into account since its increase not only results in a lower concentration of H^+^, which is the major electron scavenger, but also shifts the *E*_CB_ of minerals toward more negative values.

## 3. The Indicators of Soil Redox Activity: Redox Activity of Soil

Oxidation and reduction reactions occur instantly in soils. Soil redox abilities depend on the properties and concentration of substances contained in the so-called “soil solution”.

The indicators of soil redox activity: A straightforward indicator characterizing the oxidation and reduction ratios in soil is its reduction potential *E*_h_, which can be calculated based on redox half-reactions with the Equation (2) [83,84,85,86]:

(2) $$E_{h}=E_{h}^{⊖}+2.303\frac{RT}{nF}(\log\frac{\left\{\mathrm{Ox}\right\}}{\left\{\mathrm{Red}\right\}}+m\times \mathrm{pH}),$$

where $E_{h}^{⊖}$ [V vs. SHE] is the reduction potential under standard conditions (all activities = 1, $P_{H_{2}}$ = 1 atm (101,325 Pa), {H^+^} = 1 M), *R* is the universal gas constant [J mol^−1^ K^−1^], *T* is the temperature [K], *m* is the number of exchanged protons, *n* is the number of exchanged electrons, *F* is the Faraday constant [C mol^−1^], and {Ox}/{Red} is the ratio of the activities of oxidized to reduced species.

In addition to *E*_h_, in the “soil chemistry”, the reader may encounter two more indicators of soil redox activity, which are less known to general chemists, i.e., *r*H and pe. The redox potential of soil solutions depends on the degree of saturation with molecular hydrogen (H_2_) and the pH of the environment. The higher H_2_ concentration causes the greater reduction capacity of the solution and vice versa. The *r*H indicator is a negative logarithm of the hydrogen pressure in the soil–“soil solution” system, which demonstrates the relationship between redox and soil pH [87].

(3) $$rH≃\frac{E_{h}}{30}+2\mathrm{pH},$$

Because many redox-active elements (mainly metals) are involved in soil redox processes, pE (or pe) equal to −log{activity of electron} (Equation (4)) seems to be a more universal redox indicator [71,76,86,88].

pE = log*K* − pH, (4)

where $K=\{\mathrm{Red}\}/(\{\mathrm{Ox}\}\times {\left\{e^{-}\right\}}^{n}\times {\left\{H_{3}O^{+}\right\}}^{m})$.

Redox activity of soil: In all systems, *E*_h_, *r*H, and pE are governed by the pH and the activities of oxidized {Ox} and reduced species {Red}. (For example, *E*_h_ higher than 200 mV is usually associated with the dominance of electron acceptors in the soil, e.g., O_2_, NO_3_^−^, MnO_2_, and Fe_2_O_3_.) For complex systems such as soil, where the coverage of all components in the equations can be extremely complicated, the solution is to use the professional geochemical software [89,90].

Normal limits of pH in the environment are 4 and 9, and lower are found in acid sulfate soils, while the upper end of the pH limits is associated with water in contact with carbonate rocks. Whereas the theoretical limits of *E*_h_ are determined by water instability and the release of gases, O_2_ upon oxidation, and H_2_ upon reduction. The upper limit of *E*_h_ is defined by the oxidation of H_2_O ($2H_{2}O⇌O_{2}+{4H}^{+}+4e^{-},E^{⊖}=1.23$ V), whereas its lower limit is defined by the reduction in H^+^ ($H_{2}⇌{2H}^{+}+2e^{-},E^{⊖}=0.00$ V). The potentials of the above half-reaction depend on the pH and follow the Nernstian equations. (Importantly, major photoactive minerals (see above) remain stable in these pH and *E*_h_ ranges [71,84,86,91].)

From an agricultural point of view, *E*_h_ values within 200–750 mV are beneficial for normal plant development. The *E*_h_ value of 750 mV for soil is associated with full aerobiosis, at which there is already a violation of the correctness in plant nutrition, while *E*_h_ lower than 200 mV is associated with reducing processes harmful for plants. The potential *E*_h_ is mainly influenced by the soil moisture, pH, and microbiological reactions. Increasing soil moisture reduces the value of *E*_h_, and drying has the opposite effect. *E*_h_ fluctuates depending on the hydrologic regimes [92] and the season. For instance, in the temperate climate zone (of middle latitudes 23.5° to 66.5° N/S of the equator), it is the lowest in spring and increases in summer and autumn [87].

It seems obvious that sunlight has its share of daily and seasonal fluctuations of *E*_h_ since the photochemical redox processes occur upon sunlight. Interestingly, even the effects of soil drying by sunlight are different from those by drying in the dark [71]. Although light will generally not penetrate the soil surface deeper than 2 mm, on light-exposed soil, this depth will be sufficient to create a redox interface, especially since upward diffusion may extend the effective depth of the sunlight. The redox balance in the soil is affected by exposure to sunlight. Numerous soil components are photoactive, and their chemistry will vary significantly in sunlight compared with darkness. They include Fe(III) species, polycarboxylates, humic acids, and MnO_2_. Probably the most prevalent reactions in soil are photoredox transformations of Fe(III) and associated organic ligands [93,94,95].

The balance of redox in soil changes under the influence of light in numerous, often competing processes. These may be reversible processes such as the Fe^2+^/Fe^3+^ redox transformations, as well as irreversible, after which reaction products such as CO_2_ leave the soil environment. Importantly, environmental toxins may also participate in photochemical processes, which may lead to their degradation. Examples include the degradation of aromatic and polyaromatic hydrocarbons, aryl ketones and dioxins caused by ^•^OH radicals produced in the Haber–Weiss reaction [71,96], and photocatalytic nitrogen-oxide conversion in red soil [97,98].

## 4. Susceptibility of Organic Pollutants and Pathogenic Microorganisms to Oxidative- and Bio-Degradation: The Persistent Micro-Pollutants as a Main Challenge for AOPs

Here, we should emphasize that research on the decontamination of waters containing persistent organic pollutants has been the core of photocatalytic research for years (see, for example, a review [99]).

Thus, in addition to the research focused on antimicrobial nanomaterials to inhibit bacterial growth and destroy the cells, many photocatalytic disinfection studies have been performed involving bacteria, fungi, algae, and viruses [66,67,100,101,102,103,104,105,106,107,108,109,110,111,112,113,114,115,116]. On the other hand, in order to limit the spread of antibiotic-resistant bacterial strains, the photocatalytic degradation of antibiotics was also examined [117,118,119].

Numerous experimental and review papers have been published. Particularly worth recommending are the relatively recent reviews on the application of photocatalysis for toxicity reduction in real wastewaters [120] and the elimination of viruses and other pathogens from recreational waters, food packaging, hospital surfaces, etc. [121,122,123,124].

The recent reviews of Valeriani et al. [125,126] are particularly noteworthy. Both works were carried out according to a rigorous protocol of meta-analysis [127], demonstrating the most current trends in the antimicrobial effectiveness of innovative photocatalysts.

The persistent micro-pollutants as a main challenge for AOPs: With few exceptions, like perfluorinated and polyfluorinated substances (PFASs) [82,128], the ROS generation in photocatalysis very effectively induces oxidative decomposition of pollutants, which can even lead to their complete mineralization [8,15]. Therefore, one can hypothesize that the photocatalysis processes on the soil surface will take part in natural oxidative reduction processes occurring in soil [88] and as such may contribute to its decontamination. However, a complete mineralization (i.e., the formation of CO_2_, H_2_O, and NH_3_, exclusively) of the pollutant seems essential in many cases since degradation products have the potential to be as harmful or even more harmful toxins than the parent compounds.

The persistent micro-pollutants are frequently eliminated from the waste applying a multi-stage process where so-called advanced oxidation processes (AOPs) [2] are usually used prior to the biological stage to initially decompose the pollutants [129,130]. The micro-pollutant leftovers can appear incidentally even in the municipal waste, where the AOP pre-treatment may potentially worsen the situation due to the formation of novel toxins upon oxidation. For instance, this appears to be the case for phenylurea-derived compounds like herbicides such as *Linuron*, *Diuron*, and *Metobromuron* or the antimicrobial additive of personal care products such as *Triclocarban* [131,132,133,134,135,136,137,138,139]. The phenylurea herbicides’ production and use on beans, soybeans, tomatoes, tobacco, potatoes, flax, and sunflowers will result in their release to the environment through various waste streams. If released to the soil, the phenylurea herbicides will have moderate mobility. Volatilization of them should not be important; thus, the herbicides may be degraded on soil surfaces. Research shows that some wastewater bacteria are able to hydrolyze the urea bridge in phenylurea herbicides producing monochloroanilines and dichloroanilines [139,140,141,142]. The fate of these metabolites is not certain; however, they may slowly decompose, as well as bioaccumulate or bind to soil particles and undergo auto-oxidation [143]. This is of special importance since chloroanilines have been named “probable carcinogens” by the U.S. EPA due to their association with bladder cancer [27,28,29,144,145,146]. The environmental toxicity of *Linuron* and its metabolites had been partially eliminated with its replacement by *Metobromuron*. However, our laboratory study and computational predictions for both herbicides (*Linuron* and *Metobromuron* as well) foresees the formation of similar hazardous products upon the AOP treatment [134,147,148,149]. Among them are cyanates, e.g., isocyanatomethane (methyl isocyanate, MIC, CH_3_-N=C=O)—the toxin accused of causing nearly 3800 deaths in the Bhopal disaster [35,150,151]. Therefore, the suspicion that trace amounts of MIC could be formed during incomplete degradation of linuron-like pesticides ought to raise legitimate concerns.

Even when the situation is not particularly dramatic, peculiar products of the AOP reactions can avoid subsequent biodegradation. Moreover, such contamination may be significantly harmful or even destroy successive biological stages of waste decontamination [130,148,152].

It appears that the disinfection of wastewater is an easier process than the elimination of persistent organic pollutants. On the “molecular level” the processes of disinfection/hygienization of waste leads to decomposition of the natural, organic compounds, which are essential for pathogen survival and multiplication [153].

Individual molecules of proteins, lipids, sugars, and nucleic acids are relatively unstable and quite easily oxidized and hydrolyzed [154,155,156,157,158,159,160]. However, living cells are able to regenerate oxidative damage quite efficiently through enzymatic and non-enzymatic repair processes [161]. (It should be noted that this could lead to the selection of pathogens with greater resistance.) Therefore, one must keep in mind that the reduction in the pathogen population to a level corresponding to the requirements imposed by regulatory institutions (see [162]) will require an oxidation process of high intensity. It is unlikely that will be achievable in natural conditions because only a small portion of solar energy can be utilized in photocatalytic processes, which is evident from the comparison of *E*_BG_ values, shown in Table 1, with the well-known spectrum of sunlight (see https://www.astm.org/g0173-03r20.html, accessed 1 July 2024).

## 5. Interference of Redox Processes by Soil Organic Matter: Impact of Humic Acids on the Effectiveness of Photocatalysis In Vivo and In Vitro

Soil organic matter (SOM) is one of the key elements of carbon circulation in nature [163,164]. SOM seems to be the most valuable part of the soil from an agricultural perspective but also for growth of natural vegetation cover [87,165]. It consists of mainly humified organic debris of plants and other organisms and labile organic compounds derived from exudates of soil microorganisms and plants’ roots. Numerous functions are performed by SOM in soil, starting from physical functions (the stabilization of soil structure, water retention, and thermal properties) [165,166,167,168,169]; through chemical functions (the retention of cations, buffering capacity and pH effects, chelation of metals, and interactions with xenobiotics); and ending with the biochemical functions such as a reservoir of metabolic energy, a source of macronutrients, ecosystem resilience [165,167], or even allelopathy [170]. Interestingly, the reducing environment of humic acids promotes the formation of metallic and oxide nanoparticles in both laboratory and natural conditions [75,171,172].

From the point of view of the environment decontamination, an interesting feature is the immobilization of inorganic substances as a result of the formation of complexes with inorganic cations. For example, humified organic matter and polyvalent metal cation complexes participate in the formation of micro-aggregates with clusters and silt particles, oxides, and aluminosilicates [166]. On the other hand, the immobilization of toxic-to-plants noble metals, by humic acids of peat, in the form of metal nanoparticles was observed [171,173] and confirmed in laboratory conditions [75,174,175,176]. Unfortunately, water-soluble humic acid (HA) compounds in the disinfection processes of drinking water and wastewater are considered as precursors of highly toxic, carcinogenic, and mutagenic disinfectant by-products [150,177]. The chemistry of the processes leading to the formation of toxic derivatives of HA has been previously extensively studied and described in the basic works on radical chemistry of aromatic compounds [178,179,180]. HAs are poly-aromatic compounds that have a variety of components including quinone, phenol, catechol, and sugar moieties [181,182], with significant antioxidant properties and the ability to scavenge free radicals [182,183,184,185,186,187,188,189,190,191,192,193,194,195,196,197,198,199,200,201].

The strong inhibitory effect of natural organic matter is also a major challenge for photocatalytic water purification. This organic matter can scavenge photogenerated $h_{\mathrm{VB}}^{+}$, $e_{\mathrm{CB}}^{-}$, and radicals and occlude ROS generation sites upon adsorption. Additionally, the quantum efficiency of photocatalysis can be reduced due to the absorption of light by organic compounds, when the light quantum has too low energy to cause dissociation of chemical bonds and its absorption causes the solution to only heat up [202]. The fact that humic acids scavenge ^●^OH radicals and its precursor $h_{\mathrm{VB}}^{+}$, desired when photocatalysis is used for the degradation of humic acids, causes a decrease in the effectiveness of photocatalysis when its purpose is to degrade other pollutants [189]. A large contribution has been made to improve the quality of drinking water, thanks to the development of organic matter removal methods [203,204,205,206,207,208]. In this trend, works dedicated to increasing the photocatalysis’ efficiency counteracting the inhibitory effect of humic acids by decreasing the HA surface adsorption and mitigation of the $e_{\mathrm{CB}}^{-}$-$h_{\mathrm{VB}}^{+}$ recombination were also created [209,210,211].

## 6. The Perspectives of Photocatalysis on Soils Minerals “In Vivo”: The Limits Set by Diversity of Soils and the Environmental Conditions on the Earth

Soil is a major component of the Earth’s geosystem and constitutes the outer layer of the lithosphere (the continental crust). Soil-forming factors include climate, relief, parent material (bedrock), organisms (plants, animals, fungi, and human being), and ground water; they all interact over time [212,213,214,215].

Soil consists of not only a solid phase (minerals and organic matter) but also a porous phase (gases and water). Usually, the solid phase consists of half of the soil, thus significantly affecting the physical and chemical properties.

The continental crust is characterized by a huge variability of minerals and rocks. Because they are a soil substratum, the elements derived from weathering form soil’s chemical composition. The elements found in the continental crust are divided into three groups depending on the average occurrence (Clarke number [215]). The first group consists of elements with a high Clarke number, which constitute the main mass of rocks and soils. This group includes O, Si, Al, Fe, Ca, Na, K, Mg, C, H, S, P, and Cl [216]. They determine the geochemical properties of the landscape, mainly the conditions for the migration of other elements [217]. The second group is low Clarke number elements. Their migration depends on the conditions created by the elements of the first group. The last group is rare elements, whose content in the continental crust is lower than 0.01%.

Iron as a first group element commonly occurs in the soil. The Fe content in the continental crust is between 4.1 and 5.1%, depending on whether its mass or weight share is calculated [214,218]. Iron oxides are considered to be chemical compounds with great potential in photocatalysis. Ti is similar (0.3–0.6%, second group) [219]. These two semiconductors are quite common in soils, especially containing Fe, so there is a possibility to use the topsoil in the process of photocatalysis [200,220,221].

In addition to a photocatalyst, which is stable under given pH value and temperature conditions, for oxidation to sunlight to occur, the following are also necessary: sunlight, atmospheric oxygen, and humidity. The soil surface is exposed to sunlight in the ca. 280 to 4000 nm range [222,223], so it has the ability to initiate solar energy conversion into chemical energy and therefore to control environmental pollution and decontamination [224,225].

Experimental research on the soils’ usage in the decontamination of pollutants has been carried out several times [97,200,226]. The obtained results are promising, but are there opportunities to use this phenomenon outside the laboratory? There is no clear answer to this question yet. Under natural conditions, soil, as a complex component affected by many soil-forming factors, is characterized by significant variation of physical and chemical properties and undergoes many transformations. There are a number of crucial issues that would need to be resolved for widespread photocatalytic soil usage in vivo.

The supply of solar energy and oxygen is limited to the topsoil. In most climate zones, radiation is largely absorbed by the vegetation. In woody and shrubby vegetation zones, only a small part of the radiation reaches the soil surface [227]. The Earth’s areas with sparse vegetation are a different situation; these are primarily the desert, semi-desert, and tundra zones. The supply of solar energy is much higher because of low soil shading [228,229]. In the polar climate (tundra zone), the radiation is limited with large seasonal variability, including polar nights [230]. In deserts and semi-deserts, the supply of energy is significant and is not disturbed by cloud cover. This is a consequence of low humidity and high atmospheric pressure. In these areas the soil cover is thin or not present at all. On the surface, there are different rocks and minerals (sands, clays, etc.). In the temperate climate zone, the soil surface is covered by vegetation almost all year long. Only agricultural areas before the plant growing season (usually from October until April) are not covered by vegetation. Another issue is the variability of the soil surface. Mostly, relief is not flat, so there is a variation in the supply of energy to the surface. In the Northern Hemisphere, the northern slopes are less exposed to the sunlight than the southern slopes. That affects the soil properties, such as the thickness, moisture, and nutrient content [231]. The solar radiation depends on the height of the sun above the horizon, which varies depending on the season. For example, the sun angle for Warsaw (42° N, Poland) difference between December and June is more than 45 degrees. (To obtain an overall, global picture of the solar energy supplied to the Earth’s surface, the reader can go to the handbook [222] and to the online interactive maps on the “World Bank. Global Solar Atlas” web page [230]).

The presence of Fe oxides in the soils is a fact, but their content varies both spatially and on a global scale, as in the soil profile [232,233,234]. Fe evolution in soils is controlled both by natural factors (rock weathering, pedogenic processes driven), causing Fe transformation and translocation within and from soil (eluviation illuviation and reduction oxidation processes) [235], and by human impacts (industry and agriculture) [236].

Assuming that the photocatalysis occurs only in the surface, the presence of free oxides in the topsoil (humus horizon) is negligible. Their greatest accumulation occurs in horizons, such as *ferralic*, *nitic*, or *cambic*, which occur deeper in soil profile [237]. Hence, soils in which Fe oxides are abundant are mainly tropical and subtropical soils, such as Ferralsols and Nitisols. In the humus horizon, the content of Fe oxides is lower due to accumulation by humus, which constitutes the sorption complex of the soil.

The diversity of soils on the Earth means that the environmental conditions, such as climate, parent material, relief, and vegetation, should be included in experiments of photocatalytic properties and deeply studied in the future. The potential of the soils is huge; they can be used as the basis of more sustainable alternatives, instead of synthetic materials for decontamination of the pollutants.

## 7. Challenges and Future Perspectives

Our work on the review has shown the need for cooperation and information exchange between researchers involved in the photocatalysis field and environmental scientists. Personally, we learned a lot from each other during our writing sessions. In fact, the initiative for this review came from chemists involved in photocatalysis research (DP and JK). It happened under the influence of events related to the COVID-19 pandemic: The media reports were full of information about the estimated lifespan of the virus on surfaces made of various materials and the urgent need for systematic sterilization. Our experience with photocatalysis on the particles of iron oxides [38,39,40] was telling us that the wet surface of rusty steel could be photocatalytically active, and the virus’ lifespan on such a surface should be shorter than on stainless steel. We thought that the top layers of reach in iron oxide red soils should have similar properties. Thus, for example, the contaminated red soil exposed to sunlight should “purify” itself, and sunlit wasteland would be decontaminated by systematic plowing. These naive ideas were quickly dispelled during our internal discussion.

Therefore, we believe that for scientists who want to further develop photocatalytic decontamination, the main challenges may not be technical problems but rather establishing cooperation between specialists in various fields. It is unfortunate that we often use terms that are not understandable to our potential scientific partners. Without describing and understanding the basic topics, it is difficult to seize the opportunities and implement any applications. We could recommend seeking possibilities to conduct joint research and utilize local sources of natural minerals. This should help overcome the limitations and challenges of using soil minerals as photocatalysts, such as soil composition variability and the potential environmental impacts of large-scale applications.

## 8. Summary and Conclusions

The upper layers of the lithosphere can be a good source of unique semi-conducting materials of natural photocatalytic properties. A combination of many factors is required for the photocatalysis process to be effective. In paragraphs 4 to 6 of this work, we tried to show a realistic assessment of the conditions for effective photocatalytic decontamination in vivo and in vitro. Particular requirements such as adequate, intensive, and long-lasting sunlight; the presence of specific minerals; and moderate humidity of the soil solution mean that under natural conditions, photocatalytic processes cannot be fully effective. It cannot be ruled out that decontamination of desert and semi-desert soils can be partly attributed to photocatalysis, which can lead to the mineralization of organic matter. One can put forward the thesis that the probability that in this way, nature, without the support of technology, will cope with the pesticide residues or pathogenic microorganisms is very small. In practice, these processes call for highly advanced technical solutions. In rare circumstances, effective photocatalysis can occur spontaneously without human intervention. A forward-looking idea seems to be usage of natural photoactive minerals in new and existing technologies utilizing the photocatalytic process. The applications that are cited in [121], such as building materials using cement-based products, ceramic tiles, bituminous membranes, etc., can be good examples.

It appears that there are no negative prospects for photocatalysis development. There are also economic aspects behind the use of natural minerals: for many years to come the lower photocatalytic efficiency of natural semiconductors will be offset by their lower price.

## Figures and Tables

**Table 1:** The *E*_BG_, $E_{\mathrm{VB}}^{⊖}$, and $E_{\mathrm{CB}}^{⊖}$ energy positions at the zero point of charge (pH_zpc_) ^a^ for oxides and sulphides based on the Xu and Schoonen [26] data. The $E_{\mathrm{VB}}^{⊖}$ and $E_{\mathrm{CB}}^{⊖}$ values were recalculated to the electrochemical scale *E* (vs. SHE) = −*E*(AVS) − 4.44 V [77,78].

Mineral/Oxide	*E*_BG_/eV (nm)	$E_{\mathrm{VB}}^{⊖}$/V	$E_{\mathrm{CB}}^{⊖}$/V	pH_zpc_	Mineral/Sulphide	*E*_BG_/eV (nm)	$E_{\mathrm{VB}}^{⊖}$/V	$E_{\mathrm{CB}}^{⊖}$/V	pH_zpc_
Ag_2_O	0	0.25	0.25	11.2	Ag_2_S (*Argentite*)	0.92 (1348)	1.02	0.06	2
AlTiO_3_	3.6 (345)	2.8	−0.8	8.23	AgAsS_2_ (*Trechmannite*)	1.95 (635)	2.02	0.07	2
BaTiO_3_	3.3 (375)	3.44	0.14	9	AgSbS_2_ (*Miargyrite*)	0	0.07	0.07	2
Bi_2_O_3_ (*Bismite*)	2.8 (443)	3.19	0.39	6.2	As_2_S_3_ (*Orpiment*)	2.5 (496)	2.64	0.14	2
CdO (*Monteponite*)	2.2 (564)	2.37	0.17	11.6	CdS (*Greenockite*)	2.4 (517)	2.26	−0.46	2
CdFe_2_O_4_	2.3 (539)	2.54	0.24	7.22	Ce_2_S_3_	0	−0.85	−0.85	2
Ce_2_O_3_	2.4 (517)	1.96	−0.44	8.85	CoS	0	0.73	0.73	2
CoO	2.6 (477)	2.55	−0.05	7.59	CoS_2_ (*Catterite*)	0	1.05	1.05	1.5
CoTiO_3_	2.25 (551)	2.45	0.2	7.41	CoAsS (*Cobaltite*)	0	0.52	0.52	2
Cr_2_O_3_ (*Eskolaite*)	3.5 (554)	2.99	−0.51	8.1	CuS (*Covellite*)	0	0.83	0.83	2
CuO (*Tenorite*)	1.7 (729)	2.22	0.52	9.5	Cu_2_S (*Chalcocite*)	1.1 (1127)	1.1	0	2
Cu_2_O (*Cuprite*)	2.2 (564)	1.98	−0.22	8.53	CuS_2_ (*Villamaninite*)	0	1.13	1.13	2
CuTiO_3_	2.99 (415)	2.87	−0.12	7.29	Cu_3_AsS_4_ (*Enargite*)	1.28 (969)	1.59	0.31	2
FeO (*Wustite*)	2.4 (517)	2.29	−0.11	8	CuFeS_2_ (*Chalcopyrite*)	0.35 (3542)	0.88	0.53	1.8
Fe_2_O_3_ (*Hematite*)	2.2 (564)	2.54	0.34	8.6	Cu_5_FeS_4_ (*Bornite*)	0	0.11	0.11	2
Fe_3_O_4_ (*Magnetite*)	0.1 (12,398)	1.39	1.29	6.5	CuInS_2_	1.5 (827)	2.62	−0.38	2
FeOOH (*Goethite*)	2.6 (477)	3.24	0.64	9.7	CuIn_5_S_8_	1.26 (984)	0.91	−0.35	2
FeTiO_3_ (*Ilmenite*)	2.8 (443)	2.65	−0.15	6.3	Dy_2_S_3_	2.85 (435)	1.77	−1.08	2
Ga_2_O_3_ (*β-Ga_2_O_3_*)	4.8 (258)	3.31	−1.49	8.47	FeS (*Pyrrhotite*)	0.1 (12,398)	0.63	0.53	3
HgO (*Montroydite*)	1.9 (653)	2.59	0.69	7.3	FeS_2_ (*Pyrite*)	0.95 (1305)	1.43	0.48	1.4
Hg_2_Nb_2_O_7_	1.8 (689)	2.67	0.87	6.25	Fe_3_S_4_ (*Greigite*)	0	0.74	0.74	2
Hg_2_Ta_2_O_7_	1.8 (689)	2.7	0.9	6.17	FeAsS (*Arsenopyrite*)	0	0.57	0.57	1.5
In_2_O_3_ (*India*)	2.8 (443)	2.24	−0.56	8.64	Gd_2_S_3_	2.55 (486)	1.68	−0.87	2
KNbO_3_	3.3 (376)	2.5	−0.8	8.62	HfS_2_	1.13 (1097)	1.4	0.27	2
KTaO_3_	3.5 (354)	2.63	−0.87	8.55	HgS (*Cinnabarite*)	0	0.08	0.08	2
La_2_O_3_	5.5 (225)	3.59	−1.91	10.4	HgSb_4_S_8_	1.68 (738)	2.05	0.37	2
LaTi_2_O_7_	0	−0.54	−0.54	7.06	In_2_S_3_	0	−0.74	−0.74	2
LiNbO_3_	3.5 (354)	2.83	−0.67	8.02	La_2_S_3_	0	−1.19	−1.19	2
LiTaO_3_	0	−0.89	−0.89	7.94	MnS (*Alabandite*)	3 (413)	1.87	−1.13	2
MgTiO_3_ (*Geikielite*)	3.7 (335)	3.01	−0.69	7.81	MnS_2_ (*Hauerite*)	0	0.55	0.55	2
MnO (*Manganosite*)	3.6 (345)	2.65	−0.95	8.61	MoS_2_ (*Molybdenite*)	1.17 (1060)	1.46	0.29	2
MnO_2_ (*Pyrolusite*)	0.25 (4959)	1.64	1.39	4.6	Nd_2_S_3_	2.7 (459)	1.56	−1.14	2
MnTiO_3_	0	−0.4	−0.4	7.83	NiS (*Polydymite*)	0	0.59	0.59	2
Nb_2_O_5_ (*Niobia*)	3.4 (367)	3.55	0.15	6.06	NiS_2_ (*Vaesite*)	0	0.95	0.95	0.6
Nd_2_O_3_	4.7 (264)	3.13	−1.57	8.81	OsS_2_ (*Erlichmanite*)	0	0.3	0.3	2
NiO (*Bunsenite*)	3.5 (354)	3.06	−0.44	10.3	PbS (*Galena*)	0.37 (3351)	3.37	0.3	1.4
NiTiO_3_	2.18 (569)	2.44	0.26	7.34	Pb_10_Ag_3_Sb_11_S_28_	1.39 (982)	1.54	0.15	2
PbO (*Massicot*)	2.8 (443)	2.38	−0.42	8.29	Pb_2_As_2_S_5_	1.39 (982)	1.66	0.27	2
PbFe_12_O_19_	2.3 (539)	2.56	0.26	7.17	PbCuSbS_3_	1.23 (1008)	1.4	0.17	2
PdO	0	0.85	0.85	7.34	Pb_5_Sn_3_Sb_2_S_14_	0.65 (1907)	1.16	0.51	2
Pr_2_O_3_	3.9 (318)	2.7	−1.2	8.87	Pr_2_S_3_	2.4 (517)	1.39	−1.01	2
Sb_2_O_3_ (*Valentinite*)	3 (413)	3.38	0.38	5.98	PtS_2_	0.95 (1305)	2.04	1.09	2
Sm_2_O_3_	4.4 (282)	3.03	−1.37	8.69	Rh_2_S_3_	1.5 (827)	1.67	0.17	2
SnO (*Romarchite*)	4.2 (295)	3.35	−0.85	7.59	RuS_2_ (*Laurite*)	1.38 (898)	1.83	0.45	2
SnO_2_ (*Cassiterite*)	3.5 (354)	3.56	0.06	4.3	Sb_2_S_3_ (*Antimonite*)	1.72 (721)	2	0.28	2
SrTiO_3_ (*Tausonite*)	3.4 (365)	2.2	−1.2	8.6	Sm_2_S_3_	2.6 (477)	1.55	−1.05	2
Ta_2_O_5_ (*Tantite*)	0	−0.11	−0.11	2.9	SnS (*Herzenbergite*)	1.01 (1228)	1.23	0.22	2
Tb_2_O_3_	3.8 (326)	2.8	−1	8.5	SnS_2_ (*Berndtite*)	0	0	0	2
TiO_2_ (*Anatase*)	3.2 (387)	2.97	−0.23	5.8	Tb_2_S_3_	2.5 (496)	1.57	−0.93	2
Tl_2_O_3_ (*Avicennite*)	1.6 (775)	1.71	0.11	8.47	TiS_2_	0	0.32	0.32	2
V_2_O_5_ (*Karelianite*)	2.8 (443)	3.05	0.26	6.54	TlAsS_2_ (*Lorandite*)	1.8 (689)	1.52	−0.28	2
WO_3_ (*Tungstinite, Meymacite, Hydrotungstite*)	2.7 (459)	3.5	0.8	0.43	WS_2_ (*Tungstenite*)	1.35 (918)	1.77	0.42	2
Yb_2_O_3_	4.9 (253)	3.48	−1.42	8.15	ZnS (*Sphalerite*)	3.6 (345)	2.62	−0.98	1.7
YFeO_3_	2.6 (476)	2.46	−0.14	7.81	ZnS_2_	2.7 (459)	2.47	−0.23	2
ZnO (*Zincite*)	3.2 (247)	2.95	−0.25	8.8	Zn_3_In_2_S_6_	2.81 (441)	1.98	−0.85	2
ZnTiO_3_	0	−0.17	−0.17	7.31	ZrS_2_	1.82 (681)	1.67	−0.15	2
ZrO_2_ (*Baddeleyite*)	5 (248)	3.97	−1.03	6.7	----	----	----	----	----

^a^ pH_zpc_ for which the net adsorbed charge within the Helmholtz double layer [79,80] is equal to zero.

## Abbreviations

Allelopathy	Chemically mediated competition between plants
AOPs	Advanced oxidation processes
AVS	Absolute vacuum scale
CB	Conduction band
*E*_BG_	Band-gap energy
*E*_CB_	Energy of conduction band
$E_{\mathrm{Ox}/\mathrm{Red}}^{⊖}$	Standard reduction potential of the Ox/Red pair
*E*_r_	Reduction potential of reactant
*E*_VB_	Energy of valence band
MIC	isocyanatomethane (methyl isocyanate, CH_3_-N=C=O, CAS No. 624-83-9)
NOM	Natural organic matter
PFOSs	Perfluorinated organic surfactants
pH_zpc_	The net adsorbed charge within the Helmholtz double layer
POPs	Persistent organic pollutants
SHE	Standard hydrogen electrode
SRP	Standard reduction potential
VB	Valence band
